# New biofluid measures for TDP‐43

**DOI:** 10.1002/alz.083410

**Published:** 2025-01-09

**Authors:** Aitana Sogorb Esteve

**Affiliations:** ^1^ UK Dementia Research Institute at University College London, London, United Kingdom; Dementia Research Centre at University College London, London United Kingdom

## Abstract

Transactive response DNA‐binding protein 43 (TDP‐43) has emerged as a pivotal player in neurodegenerative diseases, particularly amyotrophic lateral sclerosis (ALS), frontotemporal dementia (FTD), and the recently described limbic‐predominant age‐related TDP‐43 encephalopathy (LATE).

Detecting TDP‐43 pathology in a minimally invasive manner is crucial for early diagnosis, monitoring disease progression and the assessment of therapeutic interventions. This talk explores recent advancements in the discovery and validation of novel biofluid measures aimed at detecting and characterising TDP‐43 pathology.

The era of proteomics has paved the way for the identification and validation of TDP‐43 biomarkers in biofluids. This presentation highlights the latest discoveries, encompassing both established and newly discovered candidates within cerebrospinal fluid (CSF) and blood.

As the field progresses, the integration of these measures into clinical practice may revolutionise our approach to neurodegenerative diseases bringing us closer to effective treatments and improved patient care.

